# Collectanea

**Published:** 1828-06

**Authors:** 


### COLLECTANEA.

Floriferis ut apes in saltibus omnia libant,
Omnia nos, itidem, depascimur aurea dicta.

### PRACTICAL MEDICINE.

*Iodine in Gouty Diseases.*—M. le Docteur GENDRIN lately addressed a letter to the Royal Institute on this subject. He thus expresses himself:

Among obstinate maladies, gout is, beyond contradiction, one of the most severe and painful. The great variety of medicines proposed for its cure sufficiently shows this. So many attempts, as yet futile, ought to inspire the greatest caution in any one who does not wish to expose himself to the chance of uselessly increasing the catalogue, already so numerous, of antiarthritic remedies. This caution, although it should lead to doubt, should not check efforts to extend the resources of art against so formidable an affection. In this persuasion, I (Dr. Gendrin) am going to mention a remedy which I have successfully used in treating gout, both to resolve chronic engorgements and the articular concretions which result from repeated attacks of this disease, and also to cure its paroxysms in the acute stage. This remedy is Iodine, the rational and proper use of which is without the least inconvenience.

I first employed it externally in chronic gouty tumors, because it had before been extolled in old swellings of the joints. Its resolvent action was so rapid, that I asked myself if it did not, in these cases, act on the nature of the disease? Following this indication, I used it against acute fits of gout, both externally and inwardly. Success in a violent fit of gout in a very strong man induced me to continue my experiments. Since that, seven patients with acute and violent gout have been completely cured by this remedy. In two only was the cure difficult. There it was necessary repeatedly to have recourse to the administration of the iodine, to prevent the paroxysms, and check their development when threatened. In all these cases, the continued use of iodine for two or three months after the complete cure of a paroxysm, has prevented its return. One patient has passed eight times when he should have had it, without its appearing. Another, five,—one, four,—and two only three, without relapse.

Of four gouty subjects with chalky concretions and chronic engorgement of the joints, two have been quite cured for more than four years, and only employed the iodine externally, but for a considerable time. One has been cured a year; and the fourth is now under treatment.

With all these patients, its action has been seconded by a regimen nourishing and slightly tonic for chronic gout, and demulcent in the acute gout.

I do not know (says M. Gendrin,) whether experience will continue to give as favorable results; but I make the present cases known that other practitioners may give the remedy a fair trial; and, if their experience of its utility corresponds to mine, that it might be generally adopted in the treatment of this disease. *(Revue Med.)*

---

*Melancholia, with Loss of Voice, cured by the actual Cautery.*—A man, aged thirty, of strong constitution and sanguineous temperament, was affected with profound melancholia and complete loss of voice. Numerous means were tried without benefit. It was then determined by Dr. Rossi to try cauterization on the vertex, by means of an iron heated to a white heat. This was done down to the bone. The next day the state of the patient was sensibly ameliorated, and answers, in a very low voice, could be got from him. As suppuration established itself, the intellectual faculties became freer; the voice alone did not improve. Purgatives were then had recourse to. The back part of the neck was covered with blisters, while the front was rubbed with ammoniacal embrocations. Internally, infusion of arnica, with camphorated ether; from time to time he was made to inspire ammonia; and at the end of two months of this treatment the health was quite restored. (*Repertorio di Torino.*)

---

*Encephalitis cured by Acupuncturation.*—G. Grassi, after being for a long time subject to frequent fits of dulness, caused by cerebral congestions, and which was removed by bleeding and purging, was seized with apoplexy, with hemiplegia, loss of voice, grinding of the teeth, irregular breathing, intermittent and sometimes scarcely perceptible pulse, without any signs of gastro-intestinal irritation. Repeated bleedings and purges brought him back to a pretty good state of health. At the end of some months all symptoms of local affection had disappeared; the pulse was regular; the voice a little returned, as well as the movements of the affected limbs in part, when Dr. Strambio proposed electro puncturation to complete the cure. Dr. Fantonelli performed this operation, which was done by inserting a needle at the lower part of the neck, on the side opposite to that affected; then another needle into the external ankle of the affected limb; a metallic wire, communicating with the two needles, was connected with a voltaic pile of five plates only. The introducing of the needles was not at all painful, but at each shock severe pains and violent contractions were induced in the muscles nearest to the needles, and chiefly in those of the affected side, (at the negative pile the pain was greatest.) After five or six shocks it was stopped, as the pain was intolerable.

The electro puncturation was repeated three times, with intervals of one day. After the first, the patient felt himself livelier, and moved more freely. At the second trial, he felt some inconvenience; and, on the third, he was seized with violent fever and symptoms of cerebral congestion. Bleedings and revulsives soon calmed this encephalitis, but his former ailments became just as they were before the trials were attempted. (*Giornale Critica di Med. Analitica, Milano.*)

---

*Diabetes Mammuné.*—In a case of "diabetes mammané," an abundant flow of milk following delivery, M. Picquet, thinking it depended on uterine sensibility, applied leeches to the vulva, and thus cured the disease.

---

*Case of Paruria Erratica, or Uroplania.* By Salmon A. Arnold, M.D. of Providence, R.I.—Maria Brenton, aged twenty-seven years, of sound constitution, generally enjoyed good health until June 1820, when she was afflicted with a suppression of the catamenia, accompanied with hæmoptysis. The medical attendants, irregular practitioners, bled her profusely every other day, and, after the system had become greatly debilitated, injudiciously administered emetics, the operation of which was succeeded by a prolapsus uteri, and a total inability to perform the function of urinary excretion. In this state she continued for nearly two years and a half without any alleviation of the disease, though for the most part of the time under the care of respectable physicians. The urine had been drawn off by the catheter, generally every twenty-four hours; but, when this was neglected, she often perspired very profusely about the lumbar region.

In September, 1822, soon after I first saw her, the bladder not having been emptied by the catheter for seventy-two hours, the urine found an outlet by the *right ear*, oozing drop by drop, and continued for several hours after the bladder had been emptied. The next day, at five o'clock P.M. the discharge from the ear again commenced, and continued about as long as on the preceding, but a larger quantity was poured out. The fluid was thrown on a heated shovel, and gave out the odour so peculiar to urine, indicating the presence of urea. The discharge was repeated daily for several days, oozing out guttatim, commencing earlier, increasing in quantity, and being discharged in less time; then twice a day, at three and seven o'clock P.M., varying but very little for some days; then three times a day, at two, nine, and eleven o'clock P.M.; and continuing subsequently four times a day, at ten o'clock A.M., and four, eight, and eleven o'clock P.M., increasing gradually in quantity, and being discharged in less time, until a pint was discharged in fifteen minutes in a stream about the size of a crow quill; then becoming more irregular, being discharged every four hours, and increasing in quantity until eighty ounces were discharged in twenty-four hours. The discharge from the ear was accompanied by a severe pain over the right eye and about the right ear, with a distressing sensation of fulness. The pain commenced early in the morning, and continued to increase in violence until after the discharge from the ear, by which it was relieved.

When the urine was not discharged at the usual period, or much diminished in quantity, the pain and distress were most excruciating, producing delirium, during which confinement was frequently necessary to prevent her from doing violence to her head. Sometimes a deficiency of the discharge would produce delirium, apparently without much pain: she would then laugh, sing, and converse very incoherently, though frequently with an unusual degree of wit and humour. At other times she would be seized with violent spasms, resembling opisthotonos; and, after continuing in this situation for a few minutes, the muscles becoming relaxed, she would heave a deep sigh, and then swoon, and remain in an insensible state for about half an hour, when she would sob, perhaps open her eyes, and, after repeatedly sighing, would again become insensible. In this condition she would frequently remain for more than twelve hours in succession. Sometimes the spasms would be unaccompanied with swooning; at one time her jaws were firmly closed for twenty-four hours, so that it was impossible to force them open, and at another time they continued closed for more than three days. The swooning would frequently be unaccompanied with spasm, and she would continue insensible for more than twenty-four hours, the pulse imperceptible at the wrist, and respiration only perceptible by the nicest observation. The sight of the right eye was soon destroyed, and frequently that of the left was so impaired that she could not distinguish any object across the room; but the latter is now entirely restored. The hearing of the right ear is much impaired, she cannot distinguish sounds with it; and there is a constant confused noise heard by her, like the roaring of a distant waterfall. For a short time previous to the discharge from the ear, there is a very audible noise, resembling that produced by water slowly dropping into a vessel, which may be heard at the distance of several feet. She has no power, excepting by an inclined position of the head, over the discharge, previously to which there is a painful sensation of fulness and a burning heat about the right ear. The next outlet the urine found was by the *left ear;* a few moments previous to which discharge a noise is heard similar to that noticed in the right ear: she cannot hear distinctly for ten or fifteen minutes before and after the urine passes off. This discharge is very irregular, sometimes three or four times a week, and then is discontinued for months. Soon after the discharge from the left ear, the urine found another outlet by the *left eye*, which commenced weeping in the morning and continued for several hours, producing considerable inflammation. It continued three days, and then ceased. A sufficient quantity of the fluid was saved to test its properties; in which, as well as in appearance, it did not differ from the discharge from the ear.

In the summer of 1824 it recommenced, and continued daily for about six weeks.

On the 10th of March, 1823, urine began to be discharged in great quantities from the *stomach*, unmixed with its contents. The greatest quantity was generally discharged in the morning before taking food, but it was frequently discharged soon after food had been taken, with which it was often entirely unmixed. This discharge has been more regular than from the left ear and eyes, but less so than from the other outlets.

On the 21st of April, the *right breast* became tense and swollen, with considerable pain, and evidently contained a fluid, a few drops of which oozed from the nipple. The swelling and pain continued about twenty-four hours, and then entirely ceased for about a week, when it again returned, and there was discharged a light yellow fluid resembling urine. On the 29th, one ounce was caught in a nipple shell, which, by analysis, was found to contain urea. This discharge has been very regular up to this time. Urine has been discharged occasionally from the *left breast*.

November 20th, 1823, the urine from the breast became milky, having the appearance of milk considerably diluted with water: this continued until December 12th, when it gradually resumed its usual colour.

May 12th, 1823, the abdomen, about the hypogastric and umbilical region, became violently and spasmodically contracted into hard lumps, and a sharp pain was felt shooting up from the bladder to the umbilicus, around which there was a severe twisting pain; in a few days subsequently a loud noise was heard, similar to that produced by drawing a cork from a bottle, and immediately afterwards urine spirted out from the *navel*, as from a fountain. This discharge has since continued, and has rarely been interrupted for many days. Nature, wearied in her irregularities, made her last effort, which completed the phenomena of this case, and established a discharge of urine from the *nose.* This discharge commenced on the 30th of July, 1823, oozing in the morning guttatim, and increasing in quantity every day until it ran off in a considerable stream. It continued daily for about two months, when it ceased until the summer of 1824, and then again commenced, and continued almost daily for some months, since when it has entirely ceased. All the fluids discharged were found, by analysis, to contain urea.

The following is the analysis of the fluid discharged from the right ear, made in New Haven, under the direction of Professor SILLIMAN: "Alkaline sulphates were indicated by muriate of barytes and acetic acid; muriates were detected by nitrate and acetate of silver, and by nitrate of mercury; phosphates were ascertained to exist by the addition of caustic, ammonia, and lime-water. Urea was obtained in the usual manner, by evaporating the fluid, and redissolving in alcohol, and again evaporating."

The urine discharged from the ear was, during the first six months, hotter than that from the bladder, producing by its heat pain in the external ear. The urine from the bladder turned black when not drawn off at the usual period, and deposited a sediment resembling black sand. Sometimes, after this black sediment was precipitated, the fluid would be of a dingy yellow colour; at other times it would be as black as ink. The urine from the bladder was frequently of a very high colour before it turned black, but often turned black, when it was as perfectly limpid as spring water; and on several occasions it has been noticed that, when it was most limpid, the largest quantity of black sediment was precipitated.

The discharges from the other outlets have occasionally all turned black. They differ, however, in one respect from the discharge from the bladder, since from the bladder it has never come off black, while it has from all the other outlets. This changing of colours in all the discharges from the different outlets, at the same time, is a most convincing proof of the identity of the fluids discharged.

Since the obstruction of the catamenia, there has been a discharge of blood, supposed to be vicarious: it has generally come on every five or eight weeks, sometimes at the regular period. For the first two years there was a discharge of blood, occasionally from the stomach and lungs; from the right and left ear, oftener from the left; from the right and left breast, more frequently from the left; from the navel, and from the nose. From the nose and the right ear, it has generally been mixed with urine, nearly three-fourths urine; from the left ear with about equal parts of urine and blood; from the stomach and lungs with the contents of the stomach and the secretions of the fauces; from the left breast and navel it has generally been unmixed with any other fluid. It was frequently fœtid; the colour always dark; it sometimes, though not generally, coagulates. When the urine was not drawn from the bladder for forty-eight hours, or longer, the quantity found in it was always less than when drawn off every twenty-four hours. Sometimes, when the urine was not drawn off from the bladder for seventy-two hours, it would be found to contain only one or two ounces. From which I was induced to believe that the discharge from the bladder was almost entirely useless, and that the functions of the system might be performed with very little disturbance, without the excretory office of the bladder. Accordingly I omitted to draw off the urine for seven days, when three ounces only were found in the bladder, during which period the discharges from all the other outlets were increased, and her health did not appear to have in the least suffered. Both the quantity of fluid drank, and the quantity discharged from all the outlets during the twenty-four hours, was for several days ascertained, and the difference found small. When the urine was drawn off from the bladder two or three times a day, the quantity discharged from all the other outlets was much decreased; from which I was induced to think that, if it should be drawn off every two or three hours, it would be prevented from passing into the system, and being discharged from the other outlets. I therefore introduced an elastic gum catheter, and directed the urine to be drawn off every two hours, but the catheter in half an hour would be filled up with a thick glutinous substance. This was frequently repeated with no better success. Numerous instruments were invented, and, when used, were all equally unsuccessful. Pessaries of every description, and other means to restore the parts to their natural situation, were used, and for a long time continued without any advantage. The quantity of urine discharged from the outlets was so great, and so much beyond what has been thought possible, that I was apprehensive there might have been some deception. To remove every doubt, I and my friend Dr. Webb, who at my request had occasionally attended her, remained with her four hours alternately, during twenty-four hours, and the quantity discharged during this time was as large as it had been for several days previous to and after this period. There has never been any doubt that these fluids, which have proved to be urine, were actually discharged from the ear and the other outlets, since the fact has been proved, day after day, by occular demonstration.

This great disturbance in the system continued to increase for nearly six months, and it was the opinion of all who saw the patient that she could not survive from day to day; after which period it gradually abated, and she is now, when the urine is freely discharged, so much relieved that she is able to walk about her room, and, during the summer of 1824, frequently rode out. The discharges from the right ear, the right breast, and navel, continue daily, but they are not so great nor so frequent as they were a year since; from the bladder the quantity is as usual; from the stomach, nose, eye, there has for some months been no discharge. (*American Journal of Medical Sciences.*)

---

*Vicarious Urinary Discharge.*—The only case which bears much resemblance to the above that we recollect having seen an account of, is the one related by Dr. Senter, in the first volume of the Transactions of the College of Physicians of Philadelphia. As that work has but a small circulation, the following condensed account of the case will no doubt be interesting.

Lucy Foster, a servant maid, æt. fifteen, fleshy and healthy looking, commenced to menstruate at the age of thirteen, and continued regular until about the latter end of May, 1785, at which period, the menses not appearing, and being exposed and negligent of her health, her mother supposed she had taken cold. On the 1st of June she was attacked with pain in her left hypochondrium, with cough, fever, oppression at her breast, with dyspnœa, and great irritability of the stomach. About the middle of July she vomited a quantity of bloody pus. She had a suppression of urine for twenty-four hours, which was relieved spontaneously.

In about two months she had recovered so far as to be able to return to her usual occupation, her menses returned, and continued well until June 1786, when her old complaints, with the exception of the suppression of the menses, returned with greater severity than before. The irritability of her stomach was so great that she rejected almost every article of nourishment or medicine that was given her. Opium afforded the most permanent relief.

On the 2d of July she was seized with a total suppression of urine, without any perceptible cause, which continued five days.

The beginning of the 6th day she was taken with a vomiting, and she brought up water, which she said tasted in every respect like urine. As her vomiting continued, she found relief in the bottom of her belly, from the swelling and great soreness she had felt for several days.

She now thought herself much better, but her vomiting recurred the next day, and continued more or less every day till the 14th of the month.

As she had discharged from her stomach every thing she ate or drank, from the time of her first vomiting, she did not suffer so much from the ischury as she did before the first evacuation. Dr. Senter prevailed upon her to let him pass the catheter into the bladder, whence he drew about three pints of urine, clear, but high coloured. Her strength was very much exhausted, and she felt great heat and soreness throughout the abdominal viscera.

For ten weeks successively, she was incapable of retaining in her stomach any thing except opium.

From this time to December, she continued with very little abatement of her distress; and, as she could lie in no other position, she was constantly supported in an armed chair, in a reclined posture, with pillows under her hips.

Whenever I omitted to draw off her water once in thirty or thirty-six hours at farthest, she never failed to vomit it up. To ascertain so extraordinary a fact beyond the possibility of a mistake on my part, or a deception on hers, I often visited her about the time I knew she must vomit if the catheter was not introduced; and I examined her bladder, found it full, hard, and tender; and sat by her till the vomiting recurred, saved the water that she brought up this way, and compared it with that I drew off, and found it the same in every respect.

In the month of January, 1787, from some cause unknown, she could not be relieved with the instrument, nor could she vomit up her urine for several days; when it passed off by the *navel* for three days successively; after which the catheter was used with the same effect as before.

About the beginning of August, a brick-coloured gravel began to pass off through the catheter, and soon became so large and plentiful that neither urine nor gravel could be completely evacuated by the instrument in its usual form.

She continued to discharge gravel this way, whenever her urine was drawn off, till the beginning of November, at which time she felt more distress than usual whenever her urine came off by vomiting, and she soon observed a gritty substance in her mouth. When I was informed of this new phenomenon, I requested her to save the urine for my inspection the next time she vomited. I compared this with what I drew off, and found it contained the same kind of gravel as that which passed the catheter.

From this period to the summer 1788, her complaints continued much the same. When her water was not drawn off, she always brought it up by vomiting, commonly attended with great pain in the head. The hypogastrium now became more tumid and tender, and her bladder appeared very much thickened, and extremely sore, even after it was evacuated. Add to this, the apparent inequality of the surface of the bladder was so great, and the tumor shifting sometimes towards the right and at others to the left inguen, according as her body was moved, that I began strongly to suspect a stone.

Through the month of September, her urine could very rarely be drawn off; for, upon the introduction of the catheter, a spasm seized the urethra and neck of the bladder, and, though the instrument appeared to pass high up into the fundus of the bladder, not more than a gill could be drawn before it stopped entirely, with a sensation of something falling down against its cervix, which she was very confident was a stone.

In the course of this month she vomited more sand than she had at any time before, and failed in strength and spirits so fast that I was apprehensive she would not live the month out. Her urethra, bladder, and external genital parts, were so extremely sore, that, for some time, it prevented my searching her for the stone in the manner I intended.

About the beginning of October I was able to introduce the sound, when I readily met with a stone, which appeared of a small size, and rather softer than urinary calculi commonly are. I repeated the examination a number of times, till I was perfectly satisfied that this was the case.

During the remainder of the fall, and principal part of the winter ensuing, the same troublesome sensation of the falling down of a stone in the bladder, upon the use of the catheter, continued, and induced the most excruciating pain and misery imaginable.

Her bowels, for the most part, were much less constipated than could have been expected, considering the frequency of vomiting, her supine situation, and the little nourishment she was able to retain upon her stomach; and during the whole of her disease, till within three months of her death, the catamenia were irregular. Sometimes they appeared every fortnight, and at others she passed the regular period for that evacuation two or three months, without having any; but it did not appear to me that her disease was much influenced by either.

Early in the spring 1789, her urine began to pass per anum, loaded with the same kind of gravel that had come away by the catheter. This gave her some respite with respect to her vomiting, though she continued to throw up more or less urine as well as gravel that way every week.

This new course of her water gave her a very troublesome tenesmus; but the stone in the bladder, as well as the pain and disagreeableness arising from the sensation of its descent, became daily less fatiguing. Her strength and spirits decayed fast, and the fever that she had before continually laboured under grew more completely hectical.

After the 13th of May, her bladder never became so much distended with urine as it had been before; and both this and the gravel now generally passed her once in twenty-four hours, either by vomiting or purging. She, however, introduced the catheter herself, and sometimes drew off her urine to the quantity of a gill.

The secretion of urine, as well as the formation of calculi, evidently diminished, in proportion to her loss of strength and the increase of the diarrhœa. The menses entirely ceased.

During the latter part of spring and autumn, she became quite paralytic at times; the frequency of vomiting increased, and she had several convulsion fits after vomiting. She grew more and more emaciated; her convulsions returned more frequently; her fever was more putrid; she at last became lethargic, and, on the 11th of August, death, which she had long and ardently wished for, put a period to a series of the most complicated and singular misery that I have ever seen since my acquaintance with disease.

The day after death, Dr. Senter examined the body in the presence of Drs. Waterhouse and Mason.

Thorax: In this cavity there was nothing appeared unnatural, except a considerable adhesion of the right lobe of the lungs to the pleura. Abdomen: The omentum was principally wasted, but not more than is commonly the case with those who die tabid: it was, however, of a dark gangrenous colour pretty generally. Stomach: This appeared very much changed from its natural colour, and in a gangrenous state, containing a semi-purulent matter of a fœtid scent. Liver and gall-bladder: There were no preternatural adhesions of the former, nor gall-stones in the latter; and their colour, &c. not unusual. Intestines: In these there were no ruptures either of their muscular coats, blood-vessels, or lymphatics, that we could discover. The villous coat was much destroyed, and the colour of the intestines darker than is common, except the duodenum, which was very much discoloured with the bile. Kidneys and ureters: In these there was no considerable deviation from a state of soundness; they were lax or flabby, but no rupture of any of their vessels or any calculi discoverable. Urinary bladder: This was in its natural situation, not the least thickened, had no sand or gravel in it, nor did it adhere preternaturally to any of the circumjacent parts; and the muscular sphincter of its neck yielded readily to the introduction of the finger from the bladder into the urethra. Uterus: In its cavity was contained about a drachm of thick, darkish, fœtid pus; but no other appearance of disease in its body. Tubæ Fallopianæ, were larger than common in virgins, and strung with several hydatids or vesiculæ, the size of a walnut, filled with a watery glutinous humour. Corpora Fimbriata, had a gangrenous appearance. Ovaria, were enlarged to the size of a small hen's egg, and contained a considerable quantity of a clear limpid fluid immediately under the first coat.

---

*Case of Hydatids of the Uterus, successfully treated by the Ergot;* by W. D. Macgill, m.d. of Hagerstown, Maryland. Communicated in a Letter, from Dr. J. W. Anderson, to Dr. Dewees.—I take the liberty of writing to you, to state, though not very minutely, the result of a case of hydatids of the uterus which came under the observation and treatment of my much respected preceptor, Dr. W. D. Magill, of this place. And I the more readily communicate this case to you, as you have expressed your intention of making use of the remedy which was employed in this instance, if an opportunity presented itself. It therefore gives me much pleasure in writing this letter, which I flatter myself will not be unacceptable, as it so completely confirms (as far as one case can do,) your sentiments in regard to the treatment of this disease.

Early last spring, Dr. M. was called to see Mrs. W., aged forty years, who had previously enjoyed good health, and had been the mother of several healthy children. She was labouring under a very painful affection of the womb, accompanied with periodical hemorrhagy, occurring once in twenty-four hours, usually in the evening, accompanied with febrile symptoms and much disturbance of the digestive functions, and which was evidently making rapid inroads upon her constitution. During its further continuance, her stomach became exceedingly irritable, so much so indeed as scarcely to retain nourishment of any description. Dr. M. addressed his remedies principally to the restraining the hemorrhagy, and obviating the excessive weakness of the stomach. But, notwithstanding, the symptoms continued to increase in violence, so much so as to endanger the life of the patient. She had become very much exhausted by the repeated loss of blood, and inability to take nourishment, (for she had now suffered for more than three months,) when the Doctor made an examination per vaginam, (after having made them frequently before without any satisfactory result,) and discovered something protruding through the os uteri, which he extracted, and found that the poor woman was labouring under hydatids of the womb. He immediately sent for the Ergot, judging from analogy that it would prove decidedly effectual in producing an expulsion of the heterogeneous mass. The event proved that he was not mistaken in his conjecture; for he had given the Ergot but a very few minutes when it began to show its specific operation upon the uterus, and soon ended in a complete evacuation of its contents. The mass of hydatids equalled in size the head of a large child at birth, and afforded a very good specimen of the disease; a part of which we have made a preparation of. The representation in your book on the Diseases of Women is admirable. The floodings immediately ceased, and did not return. The woman rapidly recovered, until she has at length attained her former health and vigour, and I believe she is again pregnant. She says her mother died of a disease exactly similar. (*American Journal of the Medical Sciences.*)

---

*Notice of the Efficacy of Tobacco in Cynanche Trachealis.* By N. Chapman, m.d.—Two years ago I was called to visit a lady in croup, whom I had frequently attended before in attacks of the same disease. Having used the ordinary remedies to a great extent without any decided impression on the case, it occurred to me that relaxation of the spasm of the larynx, which evidently existed, might be induced by tobacco, and accordingly directed the smoking of a cigar. Nausea was created in a few minutes, and I had the satisfaction to witness a subsidence of the affection, followed by an entire cure, without the interposition of any other means. Delighted with the efficacy of this very simple remedy, she has since exclusively relied on it, and in all the attacks of the disease, which have been numerous, for she is hardly ever exposed to its causes without suffering in this way, she has found it equally successful.

Every case of spasmodic croup which has subsequently come under my notice has, in the early stage to which I consider the tobacco only applicable, so readily yielded to the common measures, that I have had no occasion to give it a further trial. But, in several instances of the same condition of asthma, I have used it with great advantage.

Much discussion has taken place relative to the pathology of croup, whether it be of a spasmodic or inflammatory nature. It is probable that, whenever it suddenly comes on, it must be essentially of the former character, as some time is required to induce phlogosis, which is the result of a much slower process. No cause, however, more rapidly promotes it than spasmodic constriction, and hence no long period elapses till inflammation becomes established. That such a view is correct, seems to be confirmed by the symptoms, the treatment, and the post-mortem appearances in these cases. (*Ibid.*)

---

### PATHOLOGY.

*A curious Phenomenon resembling Electricity in a Case of Injured Spine.* By S. Colhoun, m.d.—Anderson Garden received an injury of the spine, and, when he became the subject of the following observations, was paralytic, from the umbilical region downwards, with incontinence of urine, which came on about three weeks after the accident had occurred, and continued till his death. From lying on his back, the integuments sloughed extensively. After some days sensibility returned, and also the power of motion. On the back nothing peculiar could be observed, excepting a different colour of the skin, which could be seen at the place of injury. His urine had a peculiar smell.

One morning, his sores suppurating well, and his health, though weak, better than it had been, he told me that when he touched any part of his body below the place which had been injured, which was somewhere between the second and sixth dorsal vertebræ, he felt a sudden sensation shooting from the parts touched to his toes, and not in the direction towards the head. The parts from which this peculiar sensation proceeded were those which were paralytic, and those only. The other parts had their natural feeling: the former had that numb and prickling peculiarity of sensation which results from the pressure of a nerve, or, as it is usually expressed, the patient said his limbs were asleep. The susceptibility of being stimulated by the touch had entirely left the legs when I touched him last, and I left him to see how long the excitability would be accumulating. I neglected to examine him for some time after. In five days I found that this peculiar quality not only extended to the middle of his back, but up to his head. It was sudden and convulsive: on slightly touching him, it produced no effect; but, if I pressed my finger with more violence, the sensation flew to his head ; but this was the case only when I pressed on the muscles, and particularly those of the thigh. Pressure on the tendons did not excite them. This peculiar sensibility was greatest after sleep, and, after rubbing the muscles two or three times, it left the parts entirely: those parts, however, which were not rubbed still retained it.

In the evening, I pressed upon the knee; then the sensation flew to his head and his feet. The head itself was, however, not affected. Squeezing the toes also gave him the same sensation: at this time it extended to his brain, and gave him an indescribable feeling. In this experiment, the muscles, on drawing the fingers along them, lost the power of exciting this peculiar feeling; still, squeezing the toes of the same side excited it. The skin also produced this phenomenon on being excited by pressure or rubbing.

A few days before his death this peculiarity ceased, and, as we had no opportunity of examining his body, we are entirely in the dark with regard to the nature of the injury, or the cause of the appearances above described. *(Philadelphia Journal of Medicine and Surgery.)*

---

### SURGERY.

*On a Case of Wound of the anterior Tibial Artery.*—A very interesting case of wound of the anterior tibial artery occurred some time since to the editor of this Journal, attended with phenomena exceedingly interesting in relation to the physiology and pathology of the circulation, and very well illustrating principles advanced by Professor SMITH, of Yale College, in an article on the pathology of the arteries published in the fifth Number of this Journal.

The patient, a Mr. Blodget, of Jericho, Vermont, wounded the anterior tibial artery, a little below the middle of the leg, with a narrow chisel, which entered from the outside of the leg, just anterior to the fibula, and divided many of the fasciculi of the exterior muscles which envelop the artery. Undoubtedly the artery was merely partially divided by the angle of the instrument. I infer this from the effects which resulted.

At the instant of the wound there took place a copious gush of blood, which very much alarmed the patient and friends. Dr. Hamilton, an intelligent young physician, was sent for, who, on his arrival, found no difficulty in suppressing the hemorrhage for the time by means of compression. As he had reason to believe the artery completely divided by the instrument, he presumed that, the pressure being continued, the hemorrhage would not recur. The patient continued apparently to do well, no more than the usual degree of inflammation supervening, until the end of two weeks, at which time, on attempting to make a greater effort than usual with the limb, blood gushed from the wound as furiously as when the limb was first wounded. Dr. H. was immediately called, and, on his arrival, the hemorrhage was commanded as before by employing pressure. The limb being at this time a good deal tumid, from diffusion of blood in the cellular tissue, Dr. H. presumed that the artery could not be found by searching the wound.

After this the bleeding occurred at intervals for two or three days, when I was called to the patient at Dr. Hamilton's request. We agreed that, as the wound was remote from anastomosing branches, and as the vicinity of the wound was much injected with blood, the best method would be to cut for the artery above the wound, and very near the middle of the leg. I immediately proceeded to the operation, and without much difficulty exposed the artery where it lies deeply imbedded between the tibialis anticus and flexor communis muscles. A ligature was thrown round it, not without some embarrassment, in consequence of its depth and the tension and volume of the muscles. Before tying the ligature I held my finger upon the artery, and distinctly felt its pulsations, and, in order to assure myself that the artery was included, I drew upon the two ends of the thread, and immediately the artery ceased to beat. This I repeated several times, so that there could be no doubt that the artery was secured. The ligature was then firmly tied, and immediately the pulsation, which before could be seen in the orifice of the wound, ceased entirely, and the blood also ceased to flow.

As the hemorrhage did not return, I left the house with very little apprehension as to the result. As I was many miles from home, however, I slept that night in the neighbourhood. In the morning I was much surprised at being called in haste to visit the patient on account of the return of the bleeding. On my arrival, however, it had again ceased; yet there was obviously a pulsation in the wound, similar to that which had been observed before the operation.

On applying my finger to the anterior tibial, upon the instep, (where all pulsation had ceased immediately after tying the ligature,) I was surprised to find that the artery pulsated vigorously, and, on comparing its beat with that of the opposite foot, I was still more surprised to find that it was much stronger and more full in the wounded limb than in the other. To ascertain the direction of its current, I then placed one finger upon the upper part of the artery, and, pressing it firmly so as to stop its circulation, I felt for its pulsations below, and found them still vigorous. I then reversed this, and found that the blood was passing in a full stream in the retrograde direction.

I thought it advisable, before attempting any thing further with the knife, to endeavour to prevent the recurrence of bleeding by compressing the artery upon the top of the foot. This was accordingly done, and apparently with good effect. As, however, I could not remain with the patient to witness the result, I advised Dr. Hamilton, should the bleeding recur, to cut down upon the region of the wounded artery, and endeavour to make compression upon the wounded vessel. This he was subsequently compelled to do, and with difficulty he saved the limb.

This case is particularly interesting in regard to the manner in which the circulation was kept up in the wounded limb.

It is obvious that the increased pulsation in the artery could not have been owing to an increase in the *vis a tergo* of the heart; for, were this the case, that of the other limb should have corresponded. The stream of blood received by the artery must necessarily have been smaller than usual, if the arteries be regarded as passive. The fulness of the artery, and its active beat, are to me conclusive evidence that the arteries exercise, in the circulation, a power which is independent of that of the heart, and which is influenced by the action of the capillaries in the inflamed part. (*Ibid.*)

---

*Case of Excision of a Part of the Spleen.* By W. B. Powell, M.D. of Ky. —In the latter part of May, 1826, J. F. was stabbed in the left side, about four hours previous to my seeing him. I found the wound covered with soot and flour, which the matrons present had applied to stop the hemorrhage. The patient was quite ungovernable from inebriation. After cleansing the wound, I found nearly two inches of the spleen protruded through an incision that had been made between the second and third false ribs, about four inches anterior to the spine, by a knife or large dirk, directed downwards, inwards, and forwards, to the cavity of the abdomen. Upon inquiry, I learned that the individual had eaten, as well as drank, but a short time before the riot, to an intemperate extent. Having satisfied myself with regard to the nature of the protrusion, I intended to attempt its reduction, but was prevented by discovering a slit in the protruded extremity, near one inch in length, which induced me to believe that such an act would endanger the patient's life from internal hemorrhage, though but little blood followed the wound, in consequence of the stricture by the lips of the wound. Hence, as the night was considerably advanced, having no other light than that of a very indifferent lamp, the patient being intoxicated, and having no disposition to pass a ligature and amputate upon my own responsibility, I deferred doing any thing more until nine o'clock next morning, when I was accompanied by Dr. Bennett, of Newport, Ky. At this time we both examined the case, and were satisfied that the protrusion consisted of one extremity of the spleen, in a high state of inflammation. In consultation, Dr. Bennett thought that we could institute no practice that would result in the recovery of the patient, from the complicated character of the wound, and the patient's habits of intemperance: hence, in his opinion, it was of no importance what was done, as he believed that death from peritoneal inflammation would ensue before the termination of the fourth day.

From my own views of the animal economy, the evidence of others relative to operations involving the peritoneum, and my own experiments upon inferior animals, I was induced to believe that a more favorable result might be hoped for. The patient was about thirty years of age, possessing but little nervous irritability, and had not prosecuted his habits of intemperance to the production of organic, or even manifest functional disease: consequently, I considered his system as possessing strong recuperative powers.

With such views of the case, I prepared a ligature from a slip of a tendon, taken from a beef's hock that had been some time in brine, and passed it around the stricture formed by the external incision, and then amputated the protruded portion. I then attempted to make a perfect reduction of the spleen, but found it impracticable, without the use of injurious force, to get the amputated extremity of the spleen below the diaphragm, because of the great contraction of that muscle.

Believing, as I did, that his system was competent to the removal of that portion of the spleen contained above the incision made in the diaphragm, I did not hesitate to let it remain, as it was reduced within the thoracic parietes: hence I closed the external wound, took a pound and a half of blood from his arm, put him to bed, prescribed light diet, and an absence from company.

On the third day, his pulse, skin, &c. indicated an unusual degree of constitutional excitement, when I ordered Tartris Antimonii, with the Sal. Soda, in small doses, and bread and milk poultices to the wound.

On the fourth day, healthy pus was discharged from the wound: this discharge continued three days, and then (I suppose) it was discharged internally, produced or followed by symptoms of peritoneal inflammation, which yielded to the administration of the above-named medicines, with warm fomentations and evaporating lotions to the abdomen.

After this he continued to improve, and on the fourteenth day was discharged from my care, though the wound was not entirely cicatrised. I saw him nine months after, when he informed me that the wound was perfectly healed, and that he enjoyed excellent health. (*American Journal of the Medical Sciences.*)

---

*Kidney Beans passed through the Canal of the Urethra.*—A man, about eleven months ago, introduced a kidney bean into the bladder, which produced all the symptoms of stone. Examination by the catheter gave very obscure signs of it. M. Civiale introduced his lithotriptor into the bladder, to measure the size of the foreign body: he laid hold of it, and squeezed it, proposing to extract it next day; but next day it came out of itself. It may be remarked, that the concretion which covered it was hardly the thickness of the twentieth part of a line.

---

*Application of Plates of Lead to Wounds.*—M. le Baron Yvan, head surgeon of the Hotel Royale des Invalides, in a letter to M. Reveille Parise, gives us the result of his extensive experience on the dressing of wounds by the application of thin plates of lead. 1st. The first trials were made on large chronic ulcers, of disgusting aspect, abundant suppuration, foul bottom, callous edges, and with most a tendency to erysipelas to some distance. The first day of the application, the pain was diminished, the suppuration changed its nature, inflammation yielded, and the edges in thickening also contracted.

2d. The extremities, which were covered with thick crusts, were equally wrapped up, as in elephantiasis. Baths, poultices, styrax plasters, sulphur cerate, had all failed in removing these crusts; but by means of the lead they fell off without effort, and permitted again the cutaneous transpiration.

3d. Emboldened by this success, he did not hesitate to employ the same means with sores in a state of hospital gangrene, and with decided good effect.

In conclusion, he adds that this mode of treating ulcers offers the immense advantage of diminishing pain, resisting the tendency to erysipelas, thickening the edges, modifying suppuration, in fine, of causing solid cicatrices.

---

*Reduction of a Shoulder which had been dislocated seven months.*—Professor Smith, of New Haven, has recently effected the reduction of a dislocated humerus seven yearly months after the occurrence of the injury. The patient was a young woman.

In this case no pulleys, nor any means whatever, were employed to multiply mechanically the extending force exerted upon the limb. Moderate extension was effected, and the usual motions impressed upon the extremity of the bone caused its head to glide into its socket. (*Philadelphia Monthly Journal of Medicine and Surgery.*)

---

*Reduction of a Femur which had been dislocated for three months.*—Dr. Morris, of Ohio, has recently furnished Dr. Smith, of New Haven, with the statement of a case in which, without employing any other mechanical means than the hands of assistants, the reduction of a dislocated femur was effected, after it had been for three months displaced.

There are very few, if any, cases on record, in which the use of pulleys has accomplished more than did the unassisted art of the surgeon in the above. We believe it will ultimately be admitted by all scientific surgeons, that force contributes infinitely less to the reduction of dislocated bones than that kind of art which is founded on a correct and minute knowledge of the anatomy and mechanism of the joint, and on a dexterous employment of manual force. By using the bone itself as a lever, the centre of motion being the point where the resisting muscles are inserted, a very great power can be exerted on the head of the bone, and of that kind which is adapted to the necessity of the case; for the head of the bone will be approximated to the socket at the same instant that the muscles are extended.

When the pulleys are employed, it is obviously impossible that, during their action, the direction of the force can be varied, or the position of the limb in the least changed. But we know that the position of the limb ought to vary with every degree of extension, and that the head of the bone should be suffered to obey the configuration of the parts, and the action of those muscles which greatly assist in approximating the head to the socket. In the hands of assistants, the direction of extension can be varied in a manner suited to these circumstances, and as much force can be applied as is safe or proper. The last motion, which slips the head of the bone into its place, is almost always exerted by the muscles, and the assistants generally feel a sudden jar of the limb at the instant of its reduction. To this effort of the muscles the elastic force of manual extension will yield, and suffer the limb to obey the natural effort. The pulleys, on the contrary, exert an inelastic force which will not yield, and the above advantage is in a great degree lost.

In a great many instances on record, *accident* has reduced dislocated bones as it has dislocated them. This has frequently occurred in dislocations of the

The American Surgeons will exceed ours.

femur. The force which, in these cases, draws the head into the cup, is evidently that of the muscles, which, in consequence of a favorable flexion of the limb, are made to act in the most advantageous manner.

Several cases have occurred in the hands of distinguished surgeons, in which, after the most powerful efforts with the pulleys and with hands, the bone has been accidentally replaced by the hands of the surgeon alone, when moving the limb in various directions to ascertain its position, or to overcome a fancied resistance in the capsular ligament. An accident of this kind occurred in the hands of a surgeon of pre-eminent skill and reputation in this city. These accidents should be studied with the utmost care. They certainly indicate the true means of effecting these reductions. If employed understandingly, they would, with precision and uniformity, produce those results which have been fortuitous. There is a great fault in our surgery when accident accomplishes more than art. (*Ibid.*)

---

*A Case of Dislocated Humerus, reduced ten months and a half after the Displacement.* By NATHAN SMITH, M.D. Professor of Surgery in Yale College. —This was the case of a lady in Derby, Connecticut. The humeri had both been dislocated into the axillæ by a puerperal convulsion. One was reduced by Dr. Smith at the end of seven months and a half after the accident, and has already been noticed in this Journal. It was thought not prudent to attempt the reduction of the other shoulder at that time, and another occasion did not present till the above time had elapsed. The reduction was then accomplished without the use of violent force or any mechanical apparatus. Gentle and long-continued extension was made upon the member; the knee of the surgeon was then placed beneath the axilla, and the bone being employed as a lever, the head was, without much difficulty, conveyed upward into the glenoid cavity.

The result of these cases should encourage us to attempt more frequently than is done the reduction of old dislocations. We believe that the frequency of failure in such attempts is often owing to the injudicious employment of the mechanical powers, it being presumed that the reduction of such requires the exercise of great force. (*Ibid.*)

---

*A convenient Mode of reducing certain Dislocations of the Humerus.*—Our distinguished friend, Dr. BELVILLE, of Trenton, communicated to us, verbally, a few days since, a case of dislocation of the shoulder, in which he effected the reduction in the following ingenious manner:

The patient was intoxicated and very refractory, which made it extremely difficult to effect the reduction in the usual position. It occurred to the Doctor, therefore, to place him upon the sound side on the floor, and to effect counter-extension and the confinement of the patient at the same time, by passing a folded sheet beneath the dislocated shoulder, and directing an assistant to stand on it on each side of the patient, so as to confine him to the floor. The surgeon then grasping the wrist, made extension upwards, which can be done in that position with peculiar advantage, since we can *lift* with more power than we can pull horizontally. Counter-extension being made upon the scapula by the hand of an assistant, and the usual flexions impressed upon the bone, it was without difficulty reduced to its place. (*Ibid.*)

### MATERIA MEDICA.

*Balsam of Copaiba.*—To take away the disgusting taste of this remedy, M. DUBLANC, jun. proposes to substitute for the balsam itself its volatile oil, which it is easy to extract from it. The resinous part has scarcely any power, but the oil is most efficacious He has tried this on thirty-three patients, who were all cured in five or six days. The dose of the oil may be as much as thirty-six "grammes," beginning with from four to six daily. It may be given either alone or in any aromatic vehicle, or in the form of electuary or pills with three-fourths of its weight of soap, or in lavement mixed with mucilage, or with yolk of egg. M. Dublanc uses it in this form:

℞. Syrup. Tolut. ℥ij.; Aq. Menth. distill. ℥iij.; Laudani Sydenhami m. xvj.; Alcoholis Copaibæ ℥iij. M.

The alcohol of copaiba is the essential oil distilled anew with two-thirds of its weight of alcohol, 36°, so as to render it more volatile and free from smell.

---

*Oleum Ricini.*—M. LANGIER states that the repeated employment of this oil as a purgative has twice produced on himself a pruriginous eruption, or redness and itching, at the wrists and bendings of the knees, yet the oil was neither rancid nor bitter.

